# Supplementary material for: The Combination of Predictive Factors of Pharmacokinetic Origin Associates with Enhanced Disease Control during Treatment of Pediatric Crohn’s Disease with Infliximab
Source: Pharmaceutics. 2023 Sep 30;15(10):2408. doi: 10.3390/pharmaceutics15102408 (PMC10610097; doi:10.3390/pharmaceutics15102408)

pharmaceutics

Supplementary Materials: The Combination of Predictive Factors of Pharmacokinetic Origin Associates with Enhanced Disease Control During Treatment of Pediatric Crohn's Disease with Infliximab

Marla C. Dubinsky [1,*], Shervin Rabizadeh [2], John C Panetta [3], Elizabeth A. Spencer [1], Annelie Everts-van der Wind [4] and Thierry Dervieux [4,*]

Table S1: **Predictive factors of PK origin and time to CRP based clinical remission in pediatric CD**

	Time (days)	HR	p value
**Infusion 2**			
Clearance above 0.294 L/day	110±27	ref.	
Clearance below 0.294 L/day	71±14	1.3 (0.9 to 2.0)	0.188
IFX below 20µg/mL	95±18	ref.	
IFX above 20µg/mL	69±13	1.4 (0.9-2.1)	0.140
**Infusion 3**			
Clearance above 0.294 L/day	138±28	ref.	
Clearance below 0.294 L/day	76±13	1.5 (1.0 to 2.3)	0.047
IFX below 15µg/mL	139±20	ref.	
IFX above 15µg/mL	46±12	2.3 (1.5-3.6)	<0.001
**Infusion 4**			
Clearance above 0.294 L/day	188±35	ref.	
Clearance below 0.294 L/day	68±11	2.1 (1.3 to 3.2)	0.001
IFX below 10µg/mL	142±21		
IFX above 10µg/mL	53±12	2.2 (1.4-3.3)	<0.001

Table S2: PF of PK origin either alone or combined at the end of induction and during maintenance and disease control over maintenance period.

	**parameter***	**estimates**
Time<br>and IFX >5µg/mL	θ_{pop}<br>θ_{time}<br>$\theta_{concentration}$<br>-2LL	-0.38±0.53 (p=0.473)<br>0.004±0.002 (p=0.046)<br>1.53±0.43 (p<0.001)<br>361.6
Time<br>and Clearance <0.294 L/day	θ_{pop}<br>θ_{time}<br>θ_{Cl}<br>-2LL	1.53±0.58 (p=0.008)<br>0.004±0.002 (p=0.046)<br>2.57±0.50 (p<0.001)<br>353.7 (Δ=-7.9; p=0.005)
Time and<br>PF of PK origin (IFX >5µg/mL with Clearance <0.294 L/day)	θ_{pop}<br>θ_{pop}<br>$\theta_{both\ PF\ of\ PK}$<br>-2LL	1.06 ±0.48 (p<0.001)<br>0.004±0.001 (p<0.001)<br>2.97±0.81 (p<0.001)<br>396 (Δ=-12.6; ; p<0.001)
Time and both PF of PK origin at infusion 4 (IFX >10µg/mL with Clearance <0.294 L/day) and during maintenance	θ_{pop}<br>$\theta_{both\ PF\ of\ PK\ 4th\ inf}$<br>$\theta_{time}$<br>$\theta_{both\ PF\ of\ PK}$<br>-2LL	0.35±0.50 (p=0.012)<br>2.44±0.64 (p<0.001)<br>0.002±0.001 (p=0.046)<br>2.51±0.73 (p<0.001)<br>377.8 (Δ=-23.2; p<0.001)

*Model: logit(Probability of CRP based remission)= $\theta_{pop}+\theta_{covi}*cov_i+\dots$

Figure S1: IFX concentration and Clearance at each of the Induction time point and maintenance. <u>*Panel A*</u>: infusion 2; <u>*panel B*</u>: infusion 3; <u>*panel C*</u>: infusion 4; <u>*panel D*</u>: maintenance

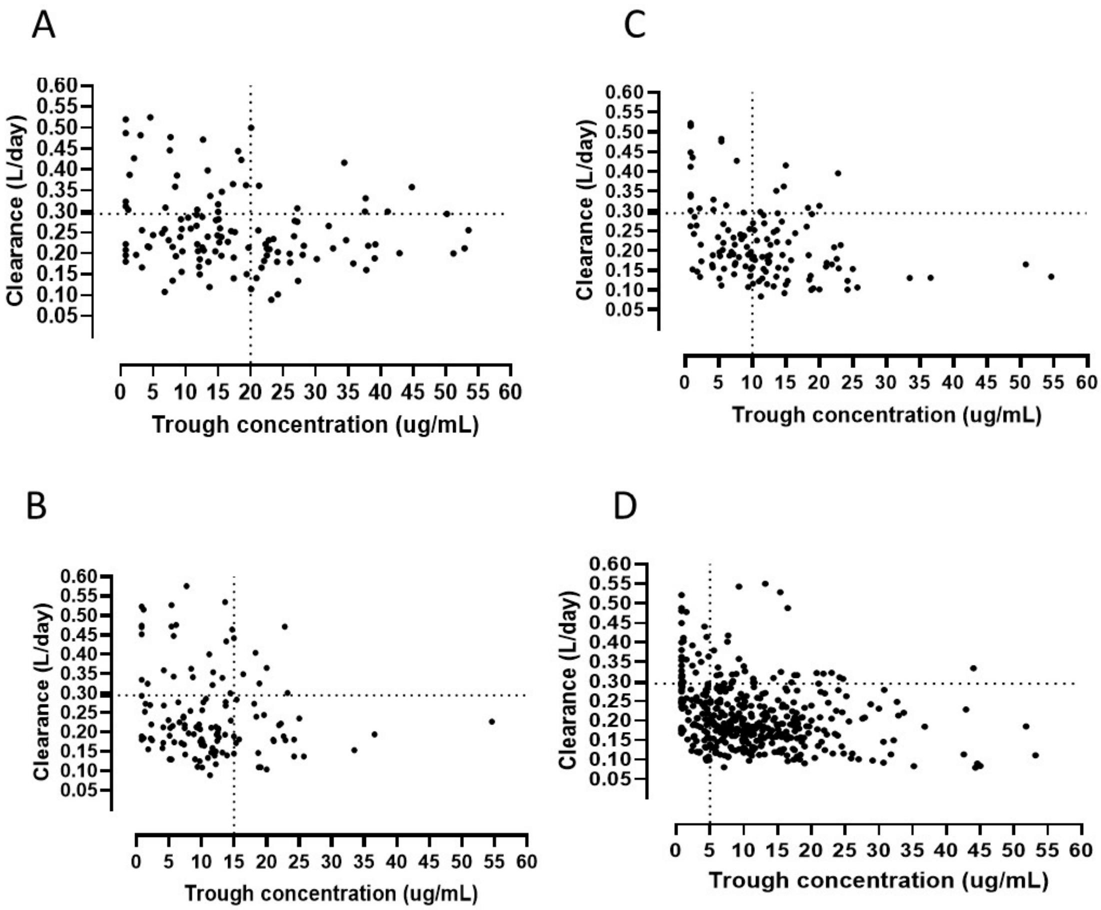

Supplement: Supplementary file 1 [file pharmaceutics-15-02408-s001.zip › pharmaceutics-2610000-supplementary.pdf]
